# Benzimidazoles Containing Piperazine Skeleton at C-2 Position as Promising Tubulin Modulators with Anthelmintic and Antineoplastic Activity

**DOI:** 10.3390/ph16111518

**Published:** 2023-10-25

**Authors:** Kameliya Anichina, Anelia Mavrova, Dimitar Vuchev, Galya Popova-Daskalova, Giada Bassi, Arianna Rossi, Monica Montesi, Silvia Panseri, Filip Fratev, Emilia Naydenova

**Affiliations:** 1 Department of Organic Synthesis, University of Chemical Technology and Metallurgy, 8 Kliment Ohridski Blvd., 1756 Sofia, Bulgaria; anmav@abv.bg; 2 Department of Infectious Diseases, Parasitology and Tropical Medicine, Medical University, 15A Vasil Aprilov Blvd., 4002 Plovdiv, Bulgaria; vuchev_d@abv.bg (D.V.); galia_val@mail.bg (G.P.-D.); 3 Institute of Science, Technology and Sustainability for Ceramics, National Research Council of Italy, Via Granarolo 64, 48018 Faenza, Italy; giada.bassi@issmc.cnr.it (G.B.); arianna.rossi@issmc.cnr.it (A.R.); monica.montesi@issmc.cnr.it (M.M.); silvia.panseri@issmc.cnr.it (S.P.); 4 Department of Neurosciences, Imaging and Clinical Sciences, University of G. D’Annunzio, Via Luigi Polacchi, 11, 66100 Chieti, Italy; 5 Department of Chemical, Biological, Pharmaceutical and Environmental Sciences, University of Messina, Viale Ferdinando Stagno d’Alcontres, 31, 98166 Messina, Italy; 6 Micar Innovation (Micar 21) Ltd., 34B Persenk Str., 1407 Sofia, Bulgaria; filipfratev@yahoo.com; 7 Department of Pharmaceutical Sciences, School of Pharmacy, The University of Texas at El Paso, 1101 N Campbell St., El Paso, TX 79968, USA; 8 Department of Organic Chemistry, University of Chemical Technology and Metallurgy, 8 Kliment Ohridski Blvd., 1756 Sofia, Bulgaria; emilia@uctm.edu

**Keywords:** benzimidazole, piperazine, tubulin modulators, anthelmintic activity, antitumor activity, LDH cellular release, cell migration, induced fit docking, molecular dynamics simulation, absolute free energy of perturbation (ABFEP), IFD-MD

## Abstract

Benzimidazole anthelmintic drugs hold promise for repurposing as cancer treatments due to their interference with tubulin polymerization and depolymerization, manifesting anticancer properties. We explored the potential of benzimidazole compounds with a piperazine fragment at C-2 as tubulin-targeting agents. In particular, we assessed their anthelmintic activity against isolated *Trichinella spiralis* muscle larvae and their effects on glioblastoma (U-87 MG) and breast cancer (MDA-MB-231) cell lines. Compound **7c** demonstrated exceptional anthelmintic efficacy, achieving a 92.7% reduction in parasite activity at 100 μg/mL after 48 hours. *In vitro* cytotoxicity analysis of MDA-MB 231 and U87 MG cell lines showed that derivatives **7b**, **7d**, and **7c** displayed lower IC_50_ values compared to albendazole (ABZ), the control. These piperazine benzimidazoles effectively reduced cell migration in both cell lines, with compound **7c** exhibiting the most significant reduction, making it a promising candidate for further study. The binding mode of the most promising compound **7c**, was determined using the induced fit docking–molecular dynamics (IFD–MD) approach. Regular docking and IFD were also employed for comparison. The IFD–MD analysis revealed that **7c** binds to tubulin in a unique binding cavity near that of ABZ, but the benzimidazole ring was fitted much deeper into the binding pocket. Finally, the absolute free energy of perturbation technique was applied to evaluate the **7c** binding affinity, further confirming the observed binding mode.

## 1. Introduction

Identifying new uses for clinically approved drugs outside the scope of their original medical indications, known as drug repositioning or drug repurposing, is an alternative and valuable drug discovery approach for pharmaceutical companies [1,2]. The associated early stages of drug development [3,4,5] are significantly faster and more cost-effective as the *in vitro* and *in vivo* screening processes have been completed; the drug’s bioavailability and absorption, distribution, metabolism, excretion, and toxicity profiles are well-established, and the formulation development is complete [6].

**Figure 1:**
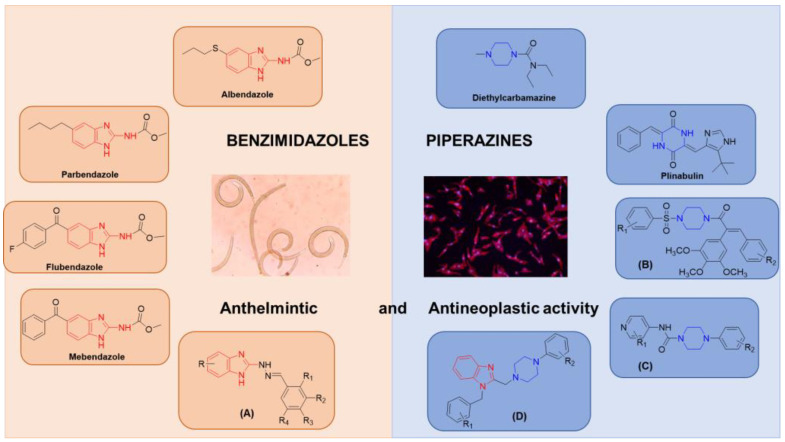
Structures of tubulin-targeting agents containing benzimidazole and/or piperazine scaffolds.

Benzimidazole anthelmintics such as albendazole (ABZ), mebendazole (MBZ), flubendazole (FBZ), and parbendazole (PBZ) (Figure 1) are long-established drugs for the treatment on human intestinal and tissue helminthiases, filarial nematode infections, trematode infections, and certain types of protozoan infections. Their long-time effective and safe utilization, coupled with their low cost and mode of action, has piqued an interest in oncologists to repurpose them as anticancer medications [7,8,9,10].

This interest arises from the observation that several features at the host–parasite interface are similar to those found at the host–tumor interface, including immune evasion, disruption of multiple signaling pathways, the presence of shared antigens, and redirection of energy and resources [11]. Furthermore, the anthelmintic effect might be related to the manifestation of anticancer properties by interfering with the tubulin polymerization and depolymerization dynamics, resulting in mitotic arrest [12]. In this regard, numerous studies, including clinical trials [13,14], have demonstrated that benzimidazole-based antiparasitic medicines exhibit remarkable antitumor activity in leukemia, melanoma, glioblastoma, prostate, breast, and colorectal cancers [15,16,17,18,19]. 

Recently, we published the synthesis of a small benzimidazolyl-2-hydrazone library (A, Figure 1) as potential microtubule-targeting agents. The new benzimidazole derivatives were initially evaluated for their *in vitro* anthelmintic activity against isolated *Trichinella spiralis* (*T. spiralis*) muscle larvae (ML). All tested compounds exhibited significant anti-*Trichinella* activities in the antinematodal bioassay, superior to the positive controls ABZ and ivermectin. Moreover, the MTT *in vitro* cell proliferation assay with the breast cancer cell line MCF-7 revealed that the tested benzimidazoles elicit low to moderate cytotoxic effects with IC_50_ values between 16.54 and 95.54 μg/mL [20].

The piperazine skeleton is a common structural element in many biologically active compounds. Piperazine, along with its various salts (hexahydrate, adipate, phosphate, edetate calcium), was among the earliest drugs employed to treat ascariasis and enterobiasis. Additionally, diethylcarbamazine (Figure 1), a piperazine derivative widely used in clinical practice, exhibits notable antifilarial activity [21]. Meanwhile, myriad piperazine-containing compounds exhibit antiproliferative effects against various cancer cells (colon, prostate, breast, lung, and leukemia cell lines) [22,23]. Notably, plinabulin (Figure 1) is a piperazine derivative that shares similarities with colchicine in terms of its ability to induce tubulin depolymerization, boasting IC_50_ values at the nanomolar scale [24]. Furthermore, several other piperazine-based agents (B, C, Figure 1) have been identified as potent antimicrotubule toxins, underscoring their potential in the realm of anticancer therapeutics [24,25,26]. 

The incorporation of piperazine fragments at the C-2 position in the benzimidazole ring has led to new compounds (D, Figure 1) with anticancer activity. These benzimidazole–piperazines hybrids exhibit noteworthy antiproliferative effects against human lung cancer (A549) with IC_50_ values of 2.8–7.8 μM [27].

The findings regarding the anthelmintic and antineoplastic properties of benzimidazole and piperazine derivatives have motivated us to explore the potential of benzimidazole compounds containing a piperazine fragment at the C-2 position the benzimidazole ring as tubulin-targeting agents. To achieve this objective, we analyzed their anthelmintic activity against isolated *T. spiralis* ML and their antiproliferative effects on human U87 MG (glioblastoma) and MDA-MB 231 (breast) cancer cells. Additionally, we conducted molecular modeling using a combination of docking, induced fit docking (IFD), and IFD––molecular dynamics (MD) methods to identify the potential binding mode. The absolute FEP+ (ABFEP) approach was employed to calculate the binding affinity of the most potent compound, exhibiting dual anthelmintic and antineoplastic properties, with tubulin.

## 2. Results and Discussion

### 2.1. Synthesis

**Scheme 1:**
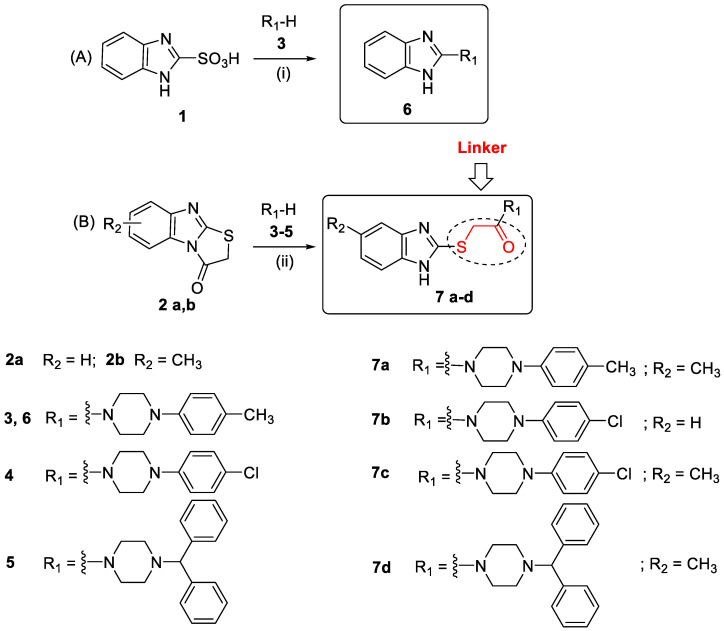
Synthetic routes (**A**) and (**B**) to generate benzimidazole derivatives containing a piperazine skeleton at the C-2 position (compounds **6** and **7a**–**d**, respectively). Reagents and conditions: (i) 160 °C, 30 min; (ii) C_2_H_5_OH, reflux 3 h.

The synthetic pipeline for preparing the desired benzimidazoles containing a piperazine fragment directly incorporated into the C-2 position of the benzimidazole ring (compound **6**) or connected via a thioethanone (-SCH_2_CO-) linker (compounds **7a**–**c**) is outlined in Scheme 1. 

The starting benzimidazole sulfonic acid **1** [28] was reacted with 1-(4-methylphenyl)piperazine **3** by heating to 160 °C for approximately 30 min, with no solvent present (Scheme 1A). The molar ratio of the reagents was 1:2 in favor of the piperazine derivate. We conducted experiments at varying temperatures. After heating the reagents for 1 h at 120 °C, thin layer chromatography (TLC) analysis indicated the absence of the targeted product. The increase in the reaction temperature to 180 °C led to undesirable charring of the mixture. 

According to US Patent Document US4093726 N-(2-benzimidazolyl)-piperazines can be prepared by replacing the chlorine atom at the C-2 position of the benzimidazole nucleus with a piperazine compound. This nucleophilic substitution is carried out by refluxing the reaction mixture in the presence of 2-methoxyethanol [29].

To prepare 1*H*-benzimidazol-2-ylthioacetylpiperazine derivatives **7a**–**d** (Scheme 1B) [1,3]-thiazolo[3,2-a]benzimidazol-3(2*H*)-ones **2a** and **2b** were reacted with the respective N-monosubstituted piperazine **3**–**5** in ethanol under refluxing conditions for 3 h [30]. All final products were purified by recrystallization with ethanol. 

### 2.2. Antihelmintic Activity

The effects of benzimidazole derivatives containing a piperazine fragment **7a**–**c** on the viability of *T. spiralis* ML were compared to that of commercial anthelmintic drugs (ABZ and ivermectin) are included here for comparison [30]. The results are presented in Table 1. The lethal effect of the tested compounds on muscle larvae was time- and dose-dependent, similar to our earlier results [31]. Notably, piperazine benzimidazoles demonstrated significantly higher larvicidal efficacy (*p* < 0.05) at 100 μg/mL after 48 h of exposure. However, after 24 h at 50 μg/mL, a substantial proportion of the parasite larvae remained viable. For instance, the most potent agent, **7c**, exhibited 92.7% activity at 100 μg/mL (equivalent to 0.249 μM) after a 48-h incubation period at 37 °C, and only 29.6% at 50 μg/mL (equivalent to 0.125 μM) after 24 h.

Significantly, 1*H*-benzimidazol-2-ylthioacetylpiperazine derivative **7a** exhibited enhanced activity compared to its analog **6**, which had a piperazine core directly bonded to the C-2 position of the benzimidazole heterocycle. More specifically, the mortality rate of muscle larvae exposed to **7a** for 48 h at 100 μg/mL was twice as high as that elicited by compound **6** (33.8%). Additionally, the substituent in the phenylpiperazine moiety influences the larvicidal effects of these compounds. Notably, the 4-chlorophenyl-substituted derivative **7c** exhibited higher activity than its 4-methylphenyl-substituted counterpart **7a**. Meanwhile, treatment of *T. spiralis* muscle ML with 100 µg/mL ABZ and ivermectin for 48 h showed 15.6% and 78.3% efficacy, respectively. In comparison, the most active piperazine derivative of the benzimidazole (compound **7c**) exhibited approximately six times greater activity than the reference drug ABZ and approximately 1.2 times more than ivermectin.

### 2.3. Cytotoxic Activity

**Figure 2:**
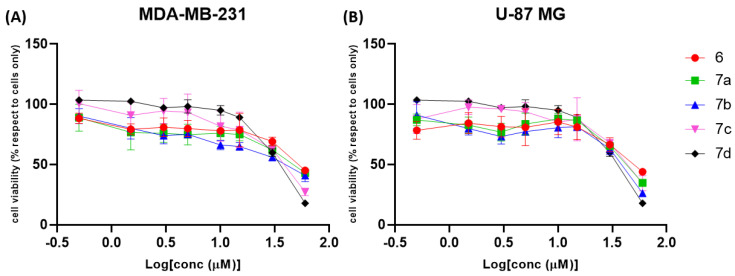
Dose-response curves. Cell viability assay conducted on tumoral MDA-MB 231 (**A**) and U87 MG cell lines (**B**) 72 h after treatment with piperazine benzimidazole compounds (**6** and **7a**–**d**) at the indicated concentrations. Dose-response curves represent the percentage (%) of cell viability ± standard deviation (SD) of treated cells.

The anti-cancer activity of the piperazine derivatives of benzimidazole was evaluated by screening their potential cytotoxic activity toward MDA-MB 231 and U87 MG cell lines compared with that of the control ABZ (Table 2). All compounds were evaluated at a wide concentration range (0.5–60 μM) to establish dose-response curves and calculate the IC_50_ (Figure 2). The IC_50_ values_,_ defined as the half maximal inhibitory concentration, and their 95% confidence interval (CI), are presented in Table 2 for both cell lines.

The tested compounds demonstrated an overall moderate level of anti-cancer activity, with variable IC_50_ values ranging from 34 to > 100 µM in both screened *in vitro* tumor models (Table 2). The leading structures in the series comprise the 1*H*-benzimidazol-2-ylthioacetylpiperazine derivatives **7b**, **7d**, and **7c**. Their IC_50_ values in both tumor cell lines were remarkably similar, falling within the 34.31–39.78 µM range compared to 83.1 µM ABZ in MDA-MB 231 cells and 38.29–42.30 µM compared to 40.59 µM ABZ in U-87 MG cells. From the series, compound **6** was distinguished with the highest cytotoxicity (Table 2). 

Analysis of cytotoxicity data revealed that incorporating the -SCH_2_CO- linker between the benzimidazole and piperazine cores, substituting the methyl group in the phenylpiperazine moiety with a chlorine atom, and introducing a methyl group at the 5-position of the benzimidazole heterocycle enhance the antineoplastic activity of the piperazine benzimidazoles against the cancer cell lines. These results are consistent with those for anthelmintic activity, lending support to the proposition that benzimidazole anthelmintics may be efficacious in combatting cancer [32]. 

### 2.4. Lactate Dehydrogenase (LDH) Release, Cell Migration, and Morphological Changes

**Figure 3:**
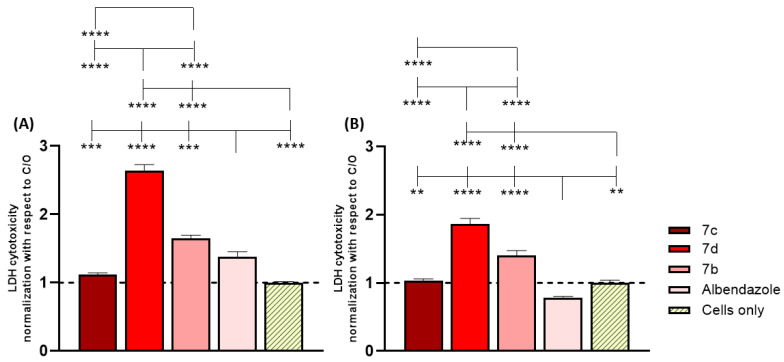
LDH cellular release. Evaluation of cellular LDH release in U-87 MG (**A**) and MDA-MB-231 (**B**) cells; statistically significant differences compared to albendazole, cells only, and among various compounds are reported; ** *p* value ≤ 0.01 *** *p* value ≤ 0.001 and **** *p* value ≤ 0.0001.

**Figure 4:**
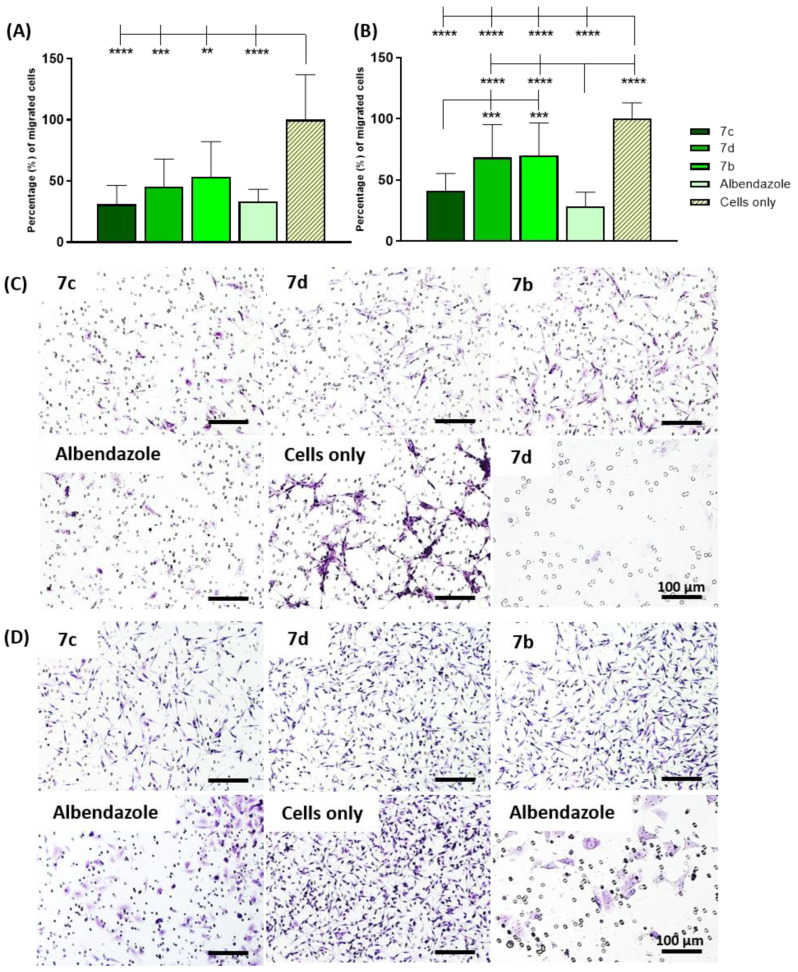
Cell migration. Quantitative analysis of U-87 MG (**A**) and MDA-MB-231 (**B**) cell migration. Statistically significant differences compared to albendazole, cells only, and various compounds are reported. ** *p* value ≤ 0.01 *** *p* value ≤ 0.001 and **** *p* value ≤ 0.0001. Representative panel of crystal violet-stained migrated U-87 MG (**C**) and MDA-MB-231 (**D**) cells. Scale bars 100 µm.

The compounds **7b**, **7c**, and **7d** were selected based on previous cytotoxicity results, and further analyses were performed to investigate their specific biological behavior. That is, the effects elicited by the compounds on LDH enzyme release, as a cytotoxic indicator (Figure 3), cell migration (Figure 4), and cell morphology (Figure 4) were evaluated. Cells were treated with the compounds at 60 µM due to the increased cytotoxic activity observed at this concentration compared to ABZ in both cell lines.

Overall, the results showed that a significant enzymatic release occurred in the presence of compounds **7b** and **7d** in both cell lines (*p*-value ≤ 0.0001) compared to cells only (Figure 3A,B). Similar results were obtained for U-87 MG cells treated with the control ABZ, showing a significant release of LDH compared to cells only (*p*-value ≤ 0.0001) (Figure 3A). Moreover, a significant release was observed for the benzimidazoles **7b** and **7d** in both cell lines (*p*-value ≤ 0.0001 in MDA-MB 231 cells; *p*-value ≤ 0.0001 and ≤0.001 in U-87 MG cells) compared to ABZ, corroborating the previously reported IC_50_ values (Figure 3A,B). LDH release was also significantly induced by compound **7c** compared to ABZ in MDA-MB 231 cells (*p*-value ≤ 0.01) (Figure 3B). Among the compounds, **7d** induced the highest enzymatic release with significant differences compared to **7c** and **7b** in both cell lines (*p*-value ≤ 0.0001). A significant difference was also observed following treatment with compound **7b** compared to **7c** in both cell lines (*p*-value ≤ 0.0001) (Figure 3A,B). Meanwhile, ABZ did not elicit significant increases in LDH release from MDA-MB 231 cells, and derivative **7c** did not increase LDH release in MDA-MB 231 or U-87 MG cells. Indeed, comparing LDH and MTT assay results can provide a good indication of the toxicity of the compounds on normal cells. Thus, based on these results, we concluded that compound **7c** is the most promising molecule because it inhibits the cancer cells similarly to ABZ and exhibits similar LDH profiles. Although the activity of **7b** and **7d** is comparable, it will likely be accompanied by more severe toxic effects.

The potential ability of the compounds to inhibit cell migration was assessed using the Transwell method (Figure 4). The quantitative analysis revealed that all the compounds significantly reduced the migration of both cell types compared to cells only (*p*-value ≤ 0.01, ≤0.001, and ≤0.0001 in U-87 MG cells; *p*-value ≤ 0.0001 in MDA-MB-231 cells) (Figure 4A,B). This suggests a specific function in inhibiting the migration of tumor cells as a key feature for tumor progression and metastasis. Treatment with ABZ caused significantly fewer MDA-MB-231 cells to migrate compared with compounds **7d** and **7b** (*p*-value ≤ 0.0001), with only 28% of cells able to cross the insert membranes compared to 70% with the other compounds (Figure 4B). Meanwhile, derivative **7c** exhibited the strongest inhibitory effect in both cell lines compared with **7b** and **7d** (*p*-value ≤ 0.001), with only 40% of MDA-MB 231 cells having migrated (Figure 4B). This trend was relatively similar in U-87 MG cells, with cells treated with compound **7c** exhibiting the lowest level of migration compared to 45% and 53% with **7d** and **7b**, respectively (Figure 4A). In U-87 MG cells, the biological activity of **7c** was also slightly higher than that of ABZ, with 30% of cells migrated compared to 33%, respectively (Figure 3A). However, no significant differences were reported between the effect of any compound and ABZ (Figure 4A,B). This further confirms that compound **7c** is the most promising candidate for further development as it exhibited better activity than ABZ in some assays and demonstrated specific mechanisms of action. In contrast, the activity of piperazine benzimidazoles **7d** and **7b** is likely due primarily to induction of generalized cell toxicity. 

The crystal violet staining of the migrated cells that crossed the membrane corroborated the quantitative analysis of compounds with both cell lines (Figure 4C,D).

**Figure 5:**
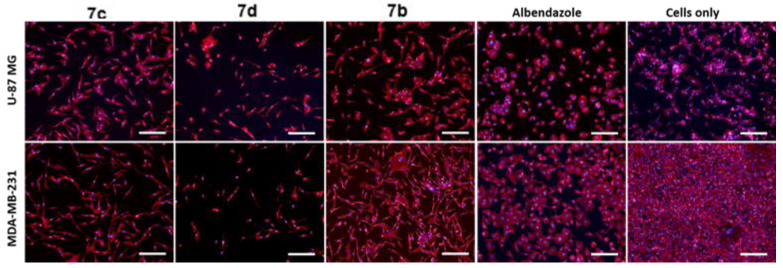
Cell morphology evaluation. Actin and DAPI staining of U-87 MG and MDA-MB-231 cells. F-actin filaments in red, cell nuclei in blue. Scale bars, 200 µm.

Morphological analysis was performed via fluorescent actin and DAPI staining. Results clearly show that ABZ alters the cell morphology of both cell types in terms of actin filament condensation, giving rise to more rounded cells. This is a morphological marker of cell death induced by the biological activity of benzimidazole and derivatives that leads to the loss of cell viability [27] (Figure 5). A morphological change was also observed following treatment with compound **7d** in U-87 MG cells, although not comparable with the distinctive round shape and cell shrinkage observed in both cell lines following ABZ treatment (Figure 5). 

### 2.5. Molecular Modelling and Absolute Free Energy of Perturbations Studies

The binding mode of benzimidazole derivatives has been investigated in several studies primarily using a simple docking approach, which is not best suited for such large and flexible binding pockets as those in β-tubulin and the space between alpha and beta tubulins. Thus, we applied more sophisticated methods to describe the binding of the lead compound **7c**. Moreover, an X-ray structure (pdb id:7dbc) was recently published of a compound that is almost identical to ABZ (only one atom difference) in a complex with tubulin, unexpectedly showing that the benzimidazole core binds more deeply into the binding pocket of β-tubulin than was previously thought. Hence, we applied this experimental structure as a template for our studies, considering that the important amino group of ABZ was replaced by a sulfur atom in our series of compounds, which may produce a different binding mode. During the structure preparation, we observed that the key ABZ binding residue in this cavity, Glu198, was protonated to Glh198. Although this result was obtained with one of the most widely used approaches (i.e., PropKa v.2.0 software), future free energy of perturbation (FEP) studies are recommended, which are outside the scope of the current study. 

**Figure 6:**
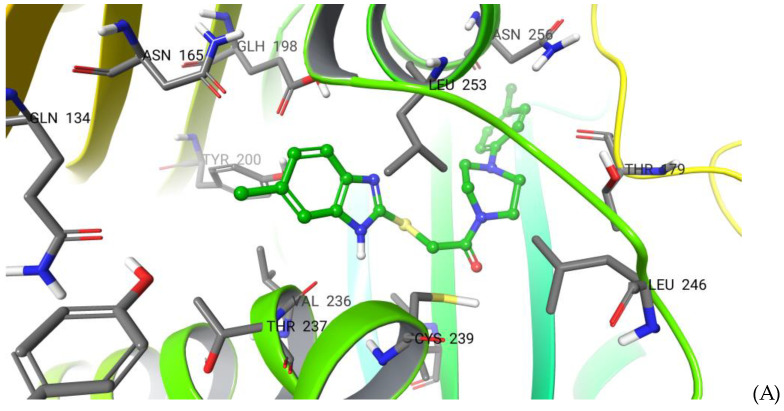

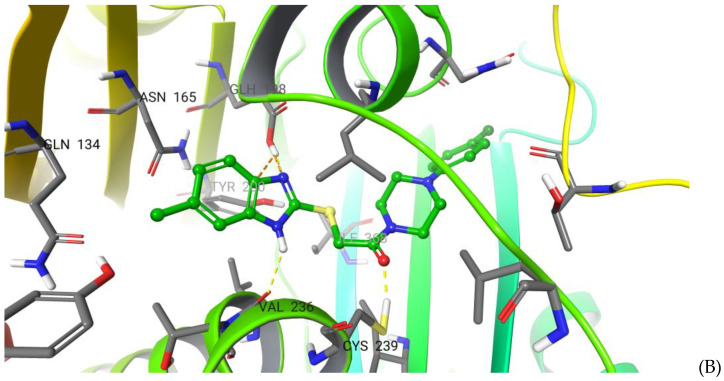
Binding mode of compound **7c** as detected by (**A**) docking and (**B**) induced fit docking (IFD).

The regular docking showed that the benzimidazole core was in almost the same position as in the ABZ-tubulin complex, surrounded by Glh198, Val236, Cys239, and Leu253. Meanwhile, the phenyl ring fit well into the colchicine binding site (Figure 6A). However, no evident key interactions were observed that explain the observed activity and higher docking score than for ABZ. Often, in docking studies, among the top-ranked poses is a conformation in which the compound is rotated approximately 180 degrees; in our case, this was observed for the phenyl ring and piperazine group in the benzimidazole ring cavity of ABZ in the β-tubulin. The generated docking poses for compound **7d** were similar to compound **7c.**

Although the docking approach has been widely used over the last 20 years, it typically achieves a 30–40% success rate in predicting the correct binding modes. This can be increased to ~60% if the flexibility of the protein residues is considered, such as in induced fit docking (IFD). Thus, we also applied IFD and found that the benzimidazole core of compound **7c** could become even more deeply situated within the β-tubulin binding site than ABZ. Moreover, several key interactions were observed. The compound forms H-bonds with Glh198 and the backbone oxygen of Val236 and is surrounded by Asn165, Tyr200, and Leu253 (Figure 6B). Additionally, sigma hole bonds are formed between the sulfur atom of compound **7c** and the Cys239 and between the chlorine atom of the phenyl ring and Thr351. Two independent classical molecular dynamics (MD) simulations of 500 ns showed that the compound seems stable in the binding cavity with an RMSD of ~1.8 Å (not exceeding 2 Å). However, the binding cavity adopted a slightly different shape, causing H-bonds and key interactions to be disrupted, including those with Glh198 and Cys239, indicating an increased likelihood of exiting in a lower free energy binding state. This further demonstrates that it is not always possible to describe the dynamic binding events using only short-term MD or combined docking and MD. 

Furthermore, we applied the recently developed approach, IFD-MD, which has a binding pose prediction rate of >90% and combines various techniques. The compound is initially docked by IFD, refined by watermaps calculations and several rescoring approaches, and the top-scored 20 poses are subjected to ten independent MD simulations. Ultimately, the top 20 scored compound conformations are selected based on their stability after ten independent metadynamic simulations and rescoring. Although these individual approaches have been applied in similar studies, their compilation in one well-defined protocol is novel. 

**Figure 7:**
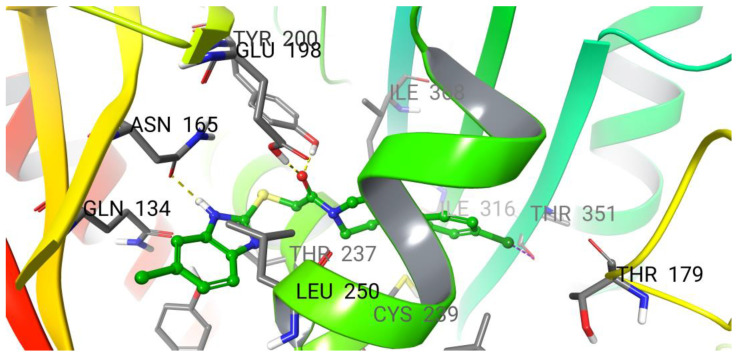
Binding mode of compound **7c** as detected by IFD-MD protocol.

Based on the IFD-MD, we obtained a unique binding conformation that differed from those obtained by IFD. For instance, the benzimidazole core was situated much deeper in the β-tubulin binding site, in an unknown binding cavity, where it forms strong interactions with the protein amino acid residues (Figure 7). Moreover, it forms H-bonds with Asn165 and hydrophobic interactions with surrounding residues, including Leu250, Tyr50, and Gln134. Meanwhile, the carbonyl group and piperazine are within the site occupied by benzimidazole in the X-ray structure of ABZ. Here, it forms strong hydrogen bonds with Glu198 and Tyr200. On the other side, the chlorine atom of the phenyl ring forms sigma hole bonds with Thr351. This is a unique tubulin inhibitor binding mode, indicating that the occupation of this site in the β-tubulin cavity by inhibitors is effective and can be optimized in future studies. For instance, the replacement of the methyl group with a more charged substituent may lead to improved binding.

Finally, we employed the absolute free energy of perturbations (ABFEP) approach to calculate the free energy of compound **7c** binding and certain pose predictions detected by different docking approaches. ABFEP calculations are the most physically rigorous method to assess the binding affinity of one compound for its receptor without having any knowledge in advance or approximations, such as the scoring function in docking or the MM/PBSA approach used after MD simulations. Meanwhile, the relative FEP predictions (RBFEP) have become one of the most accurate methods for predicting changes in the binding affinity between two similar compounds. As such, given the relatively less needed than ABFEP compu-tational resources, RBFEP is used routinely in academia and the pharmaceutical in-dustry. Nevertheless, ABFEP has been under intensive development and testing, lead-ing to its increased use following a massive decrease in the time required to complete the calculations based on new GPU units, improvements in the force fields, and many other factors. 

The ABFEP approach still depends on the initial structure and is limited by insufficient sampling time. However, this primarily impacts its ability to accurately predict the absolute free energy of binding without influencing its capacity to rank compounds based on their binding affinity. In fact, it has demonstrated superior scoring potential among various other methods. In the case of tubulin, we expected that only the ABZ binding site would be suitable for ABFEP calculations as the other regions of this large binding pocket are highly flexible, containing primarily loops. Accordingly, this region will suffer from the so-called protein reorganization effect. This effect results from the inability to sample all conformations/flexible regions of the loops and the application of only the state visible in the crystal structure. Hence, protein reorganization leads to a significant overestimation of the absolute free energy. We, therefore, ran test calculations for the ABZ derivatives and colchicine. For ABZ, we obtained a reasonable ∆G value of −8.9 kcal/mol, which was a. less than 2 kcal/mol difference from the experimentally obtained value of −7.1 kcal/mol. Thus, we concluded that it could be used for compounds that bind in this site. However, the colchicine binding site is not suitable for such calculations, as a highly overestimated value of ΔG = −15.1 kcal/mol was obtained for this inhibitor. This does not mean that ABFEP will exhibit poor performance for this site in ranking the compounds but rather highlights the need for additional assays, which are outside the scope of this study. 

For compound **7c**, we used the best pose obtained by IFD-MD (Figure 7) and a pose in which the molecule was rotated 180 degrees, i.e., the phenyl ring was situated near Glu198. We obtained ∆G values of −10.3 kcal/mol and +2.4 kcal/mol for these poses, respectively. Based on these independent calculations, we can conclude that the binding mode obtained via IFD-MD is correct and describes experimentally observed activity. The other binding mode suggested in the above approaches and by the docking experiments had a positive free energy of binding and, thus, cannot represent a binding mode of compound **7c**. We observed a good convergence during all simulations. The free energy of binding of compound **7c** in a conformation obtained using only docking had a ∆G value of −2.5 kcal/mol, whereas that obtained based on the IFD was ∆G = −5.6, indicating that these are transition states that occur along the path to those detected by IFD-MD or are inaccurate binding predictions. The binding mode detected by IFD-MD is the most likely. 

These results demonstrated the high level of prediction of the ABFEP approach compared to the empirical scoring docking functions, which are often unable to distinguish or accurately score the different poses. Moreover, our calculations provide additional confirmation that the combination of IFD-MD and FEP is a reliable approach, as the former relies primarily on the compound stability to determine the correct pose, while the latter is useful for calculating the binding affinity. We hope these results will also be of interest from a computational point of view, demonstrating the advancement of FEP calculations. Further comparison of ABFEP results obtained by different force fields and protocols are helpful but are outside of the scope of this study. 

Based on these results, we can propose structure–activity relationship (SAR) analysis and possible further optimization of the series of compounds. The *in vitro* results show that compounds **6** and **7a** are less active, whereas the other compounds in this small series have relatively similar activities. Combined with the binding pose detected by IFD-MD, we can conclude that R_1_ and R_2_ substituents make significant contributions to the altered activity. The methyl group at the R_2_ position increases the activity as it occupies the newly detected binding pocket; thus, substituting hydrogen in this position in compound **6** leads to a significant decrease in activity compared with compound **7a**. However, substituting the methyl group with chlorine in the phenylpiperazine moiety, which formed sigma hole H-bonds with Thr351, leads to an increase in the activity in compound **7c**, compared to **7a**. Meanwhile, a chlorine atom substitution at R_1_ with larger hydrophobic groups, as in compound **7d**, does not significantly improve the activity. Based on the obtained binding mode one can conclude that more efficient substitutions at position R_1_ can further increase the activity. Such substituents can provide more stable interactions with the charged tubulin residues in this portion of the binding pocket. A FEP+ guided lead optimization project in this direction is underway in our lab. 

## 3. Materials and Methods

### 3.1. General Procedures

Piperazine derivatives 1-(4-methylphenyl)piperazine **3**, 98%, 1-(4-chlorophenyl)piperazine **4**, 98%, 1-(diphenylmethyl)piperazine **5**, ≥98%, were purchased from Sigma–Aldrich (Steinheim, Germany). The organic solvents were obtained from Alfa Aesar (Heysham, UK) and Sigma–Aldrich (Steinheim, Germany) and were used without additional purification or drying. All solvents used for spectroscopy measurements were of spectroscopy-grade purity. The 1*H*-benzimidazole-2-sulfonic acid **1** was synthesized by oxidation of 1*H*-benzimidazol-2-yl-thiol and 5-methyl-1*H*-benzimidazol-2-yl-thiol, respectively with KMnO_4_ in 25% water solution of sodium hydroxide according to our previous work [28]. The synthesis of [1,3]thiazolo[3,2-a]benzimidazol-3(2*H*)-one **2a** and 6(7)-methyl-[1,3]thiazolo[3,2-a]benzimidazol-3(2*H*)-one **2b** was carried out pursuant to the synthetic procedure reported previously [33]. The reactions were controlled by TLC on pre-coated silica gel plates ALUGRAM SIL G/UV254, 0.20 mm thick (Macherey-Nagel, Düren, Germany) with a 3:1 mixture of benzene and methanol as the mobile phase. Spots on TLC chromatograms were visualized under UV light. The melting points (mp) were measured using an Electrothermal AZ 9000 3MK4 apparatus and were uncorrected. The ^1^H and ^13^C NMR spectra were recorded on a Bruker Avance 600 MHz spectrometer at room temperature (303 K) using CDCl_3_ as a solvent. Chemical shifts (δ) are reported in parts per million (ppm). Coupling constants J are given in Hertz (Hz), and spin multiplicities are given as singlet (s), broad singlet (br s), doublet (d), doublet of doublets (dd), triplet (t), triplet of doublets (td) and multiplet (m). The microanalyses for C, H and N were performed by the PerkineElmer elemental analyzer. 

### 3.2. Synthesis

#### 3.2.1. General Procedure for the Synthesis of Compounds **6**

1*H*-Benzimidazol-2-yl-sulfonic acid **1** (0.015 mol) and corresponding piperazine **3**–**7** (0.030 mol) were subjected to heating for 30 min at 160 °C. Subsequently, the resulting molten mixture was allowed to cool and then diluted with chloroform. The chloroform layer was separated and further processed by extraction with a 10% aqueous NaOH solution, followed by thorough washing with water and drying using anhydrous Na_2_SO_4_. After concentration, the target compound was precipitated, and the obtained solid was filtered and recrystallized with ethanol.

#### 3.2.2. General Procedure for the Synthesis of Compounds **7a**–**d** [30]

A mixture containing [1,3]thiazolo[3,2-a]benzimidazol-3(2*H*)-one **2a**–**b** (3.16 mmol) and the corresponding piperazine **3**–**5** (3.79 mmol) was refluxed in 12 mL of ethanol for 3 h. Afterward, the solvent was removed under reduced pressure, and the resulting oily product was washed with water to eliminate excess piperazine. Upon the addition of ethanol to the oil, compounds **7a**–**d** crystallized out.

#### 3.2.3. Compound Data

2-(4-((4-Methylphenyl)piperazin-1-yl)-1*H*-benzimidazole (**6**)

White powder (68 %), mp 205–207 °C, Rf = 0.52; ^1^H NMR (600 MHz, CDCl_3_): 10.841 (bs, 1H, NH), 9.28 (s, 1H), 7.63 (dt, 2H, *J* = 8.6 Hz), 7.28 (dd, 2H, *J* = 7.4 Hz, *J* = 40.3 Hz), 7.11 (d, 1H, *J* = 8.2 Hz), 6.95 (m, 2H), 3.35 (s, 3H), 2.50 (m, 6H), 2.25 (s, 3H); Anal. Calcd. for C_18_H_20_N_4_: C, 73.94; H, 6.89; N, 19.16; Found: C, 73.75; H, 6.98; N, 19.26.

The data for compounds **7a**–**d**, published previously [30], are incorporated herein for reference.

2-({2-[4-(4-Methylphenyl)piperazin-1-yl]-2-oxoethyl}thio)-5(6)-methyl-1(*H*)-benzimidazole (**7a**)

White powder (66%), mp 180–182 °C, Rf = 0.68; ^1^H NMR (CDCl_3_): 7.39 (d, 1H, J = 8.2 Hz), 7.27 (d, 1H, J = 1.37 Hz), 7.05–7.09 (m, 2H), 7.00 (dd, 1H, J = 1.4; 8.2 Hz), 6.79–6.85 (m, 2H), 4.08 (s, 2H), 3.60–3.79 (m, 2H), 3.09–3.15 (m, 4H), 2.44 (s, 3H), 2.28 (s, 3H); ^13^C NMR (CDCl_3_): 167.5, 148.6, 148.4, 139.0, 137.7, 132.1, 130.3, 129.7, 123.7, 117.0, 114.1, 113.9, 49.9, 49.7, 46.3, 42.3, 34.0, 21.5, 20.4; Anal. Calcd. for C_21_H_24_N_4_OS: C, 66.29; H, 6.36; N, 14.72; Found: C, 66.17; H, 6.45; N, 14.82.

2-({2-[4-(4-Chlorophenyl)piperazin-1-yl]-2-oxoethyl}thio)-1*H*-benzimidazole (**7b**)

White powder (72%); mp. 187–188 °C, Rf = 0.50; ^1^H NMR (CDCl_3_): 7.49–7.54 (m, 2H), 7.18–7.26 (m, 4H), 6.78–6.83 (m, 2H), 4.16 (s, 2H), 3.69–3.85 (m, 4H), 3.09–3.15 (m, 4H); ^13^C NMR (CDCl_3_): 167.9, 149.2, 149.1, 139.2, 129.1, 125.8, 122.4, 117.9, 114.3, 49.5, 49.2, 46.3, 42.2, 33.8; Anal. Calcd. for C_19_H_19_ClN_4_OS: C, 58.98; H, 4.95; N, 14.48; Found: C, 58.80; H, 5.06; N, 14.55.

2-({2-[4-(4-Chlorophenyl)piperazin-1-yl]-2-oxoethyl}thio)-5(6)-methyl-1*H*-benzimidazole (**7c**)

White powder (68%); mp. 172–174 °C; Rf = 0.73; ^1^H NMR (CDCl_3_): 10.78 (br s, 1H, NH), 7.38 (d, 1H, J = 7.9 Hz), 7.26 (br s, 1H), 7.18 (m, 2H), 7.18 (m, 2H), 6.96–6.99 (d, 1H, J = 7.9 Hz), 6.74 (m, 2H), 4.13 (s, 2H), 3.59–3.74 (m, 4H), 3.04 (m, 4H), 2.39 (s, 3H); ^13^C NMR(CDCl_3_): 167.4, 149.1, 148.5, 139.0, 137, 132.2, 129.0, 125.5, 123.8, 117.8, 114.1, 113.8, 49.3, 49.0, 46.0, 42.1, 34.1, 21.6. 

2-{[2-Oxo-2-(4-benzhydrylpiperazin-1-yl)ethyl]thio}-5(6)-methyl-1*H*-benzimidazole (**7d**)

White powder (57%); mp. 149–151 °C; Rf = 0.60. ^1^H NMR (CDCl_3_): 7.01–7.42 (m, 13H), 4.25 (s, 1H), 3.95 (s, 2H), 3.59–3.72 (m, 4H), 2.44 (m, 7H); ^13^C NMR(CDCl_3_): 167.6, 148.6, 141.8, 139.0, 132.0, 128.6, 127.7, 127.2, 123.6, 113.9, 75.7, 51.6, 51.2, 46.6, 42.5, 33.7, 21.5; Anal. Calcd. for C_27_H_28_N_4_OS: C, 71.02; H, 6.18; N,12.27; Found: C, 70.15; H, 6.09; N, 12.19. 

### 3.3. Anthelmintic Study

According to previously established protocols [20,30], the *in vitro* larvicidal activity against *T. spiralis* was evaluated for the two groups of piperazine-containing benzimidazoles **6** and **7a**–**d**. 

In these experiments, 100 larvae were suspended in 1 mL of physiological solution and exposed to the benzimidazole derivatives at concentrations of 50 and 100 μg/mL, dissolved in dimethyl sulfoxide (DMSO) (Steinheim, Germany, Sigma–Aldrich). Commercial drugs ABZ and ivermectin were used as positive controls. Larval viability was assessed through direct microscopy after incubation at 37 °C for 24 and 48 h, respectively. The effectiveness percentage of the compounds was determined by calculating the average value from three separate experiments.

*Trichinella spiralis* encapsulated ML were sourced from the National Centre for Infectious and Parasitic Diseases, Department of Parasitology and Tropical Medicine in Sofia, Bulgaria.

### 3.4. In Vitro Antineoplastic Study

The piperazine derivatives of benzimidazole were subjected to an initial *in vitro* screening to test their biological activity toward two different types of tumors compared to the standard ABZ as a control group. Specifically, the glioblastoma U-87 MG and the breast adenocarcinoma MDA-MB-231 cell lines were used. The compounds were first reconstituted in DMSO at 1 mg/mL before being diluted in culture media to achieve the desired concentrations. In detail, the cell viability analysis was conducted after 72 h of incubation with a wide range of concentrations (0.5, 1.5, 3, 5, 10, 15, 30, and 60 µM) of each compound to calculate the IC_50_. The negative control consisted of cells without the compounds. Additionally, a more comprehensive assessment of cell morphology, LDH release, and cell migration was carried out on both cell lines after a 72-h incubation with compounds **7b**, **7c**, and **7d** at 60 µM.

#### 3.4.1. Cell Culture

The human glioblastoma cell line U-87 MG (ATCC^®^ HTB-14™) and human adenocarcinoma cell line MDA-MB-231 (ATCC^®^ HTB132™) were purchased from American Type Culture Collection (ATCC) (Manassas, VA, USA). U-87 MG cells were cultured in MEM alpha medium (Gibco, Grand Island, NE, USA) supplemented with 10% fetal bovine serum (FBS, Gibco) and 1% penicillin/streptomycin mixture (pen/strep, 100 U/mL-100 µg/mL, Gibco). The MDA-MB-231 cell line was cultured in RPMI medium with 10% FBS and 1% pen/strep. Cells were cultured at 37 °C under controlled humidity and 5% CO_2_ atmosphere conditions. Cells were detached from culture flasks by trypsinization and centrifuged. The cell number and viability were determined by Trypan Blue Dye Exclusion test and all cell handling procedures were performed under a laminar flow hood in sterility conditions. 

#### 3.4.2. MTT Cell Viability Assay and IC_50_ Calculation

To calculate the IC_50_, the cell viability of both cell lines was determined by MTT assay. Both cell lines were seeded at a density of 25 × 10^3^ cells/cm^2^ in 96 well plates. On the following day, the compounds were added to the cells at the required concentrations, and the MTT assay was conducted after a 72-h incubation, following the manufacturer’s instructions. In summary, the MTT reagent [3-(4,5-dimethylthiazol-2-yl)-2,5-diphenyltetrazolium bromide] at a concentration of 5 mg/mL was dissolved in phosphate-buffered saline 1× (PBS 1×). The cells were incubated with the MTT solution for 2 h under conditions of 37 °C, 5% CO_2_, and controlled humidity. Subsequently, the culture medium was removed, and formazan crystals were dissolved by adding DMSO. After 15 min of incubation with gentle stirring, the absorbance of formazan was read at 570 nm measured using a Multiskan FC Microplate Photometer from Thermo Fisher Scientific. The absorbance values were directly proportional to the number of metabolically active cells in each well. One experiment was carried out and a biological triplicate for each condition was performed.

#### 3.4.3. LDH Release

The cellular release of LDH was assessed by a CyQuant LDH Fluorescence Cytotoxicity Assay (Invitrogen, Waltham, MA, USA). Briefly, both cell lines were seeded at a density of 25 × 10^3^ cells/cm^2^ in 96 well-plates. The next day, the compounds were added to the cell culture. At 72 h, 50 µL of each sample medium was transferred to a new 96 well-plate in triplicate and the assay was performed following the manufacturer’s instructions. Briefly, 50 µL of Reaction Mixture was added to each sample well, mixed and incubated at room temperature for 10 min in the dark. Then, 50 µL of Stop Solution was added to each sample well, mixed, and the fluorescence was read with the Fluoroskan™ FL Microplate Fluorometer at an excitation of 544 nm and emission of 590 nm. The data were normalized to cells only. One experiment was performed, and biological triplicates were carried out for each condition. 

#### 3.4.4. Cell Migration Evaluation

The cell migration of both cell lines was analyzed using Transwell inserts for 24 well plates with 8.0 µm pores (Corning, Somerville, MA, USA) following the manufacturer’s instructions. Briefly, MDA-MB-231 and U-87 MG cells were seeded at a density of 15 × 10^4^ and 76 × 10^3^ cells/cm^2^ respectively in the top chamber of the insert. After 24 h, the compounds were added to the cells in the top chamber of the insert under serum-free conditions and 20% FBS was used as a chemoattractant in the lower chamber. At 72 h, the cells that failed to migrate through the membrane were removed using a wet cotton swab. The cells that colonized the lower surface of the membrane were fixed in 4% (*w*/*v*) paraformaldehyde (PFA), permeabilized in 0.1% (*v*/*v*) Triton-x 100 (Sigma) in PBS 1x and stained with 0.5% (*w*/*v*) crystal violet solution (Sigma). A total of nine images per membrane were randomly acquired with the Optical Microscope and cells were counted by ImageJ Software (V 1.53T). One experiment was carried out and two biological replicates were performed for each condition. 

#### 3.4.5. Cell Morphology Evaluation

The cellular morphology was evaluated on both cell lines by Actin and DAPI staining following the manufacturer’s instructions. Briefly, the cells were seeded at a density of 25 × 10^3^ cells/cm^2^ in 96 well-plates. The next day, the compounds were added to the cell culture. At 72 h, the cells were fixed in 4% PFA, permeabilized in PBS 1X with 0.1% (*v*/*v*) Triton X-100 and stained with Actin red 555 Rhodamine Phalloidin ready probes (Invitrogen) and DAPI (4′,6-diamidino-2-ylindole, dihydrochloride) (Invitrogen), to stain F-actin filaments and cell nuclei, respectively. The images were acquired using an Inverted Ti-E Fluorescent Microscope (Nikon, Tokyo, Japan) with TRITC and DAPI filters. One experiment was carried out and two biological replicates were performed for each condition.

#### 3.4.6. Statistical Analysis

The statistical analysis was performed by GraphPad Prism Software (8.0.1 version). IC_50_ was calculated as Log(inhibitor) versus normalized response as mean percentage of live cells compared to cells only. 

The results of cell migration analysis were reported in the graphs as mean percentage (%) of migrated cells with respect to cells only ± standard deviation and were analyzed by one-way analysis of variance (one-way ANOVA) and Dunnett’s and Tukey’s multiple comparisons tests. 

The results of LDH release were reported in the graphs as mean normalized to cells only ± standard deviation and were analyzed by one-way ANOVA and Tukey’s multiple comparisons test. * *p* value ≤ 0.05, ** *p* value ≤ 0.01, *** *p* value ≤ 0.001, **** *p* value ≤ 0.0001. 

### 3.5. Molecular Modelling and Absolute Free Energy of Perturbation Calculations

All modeling studies were performed by Schrodinger suite version 2023-3 software [34]. The protein structure was downloaded from protein data bank (pdb) [35]. We used the crystal structure of tubulin complexed ABZ derivative (pdb id: 7DBC). The X-ray structure preparation for subsequent modeling was conducted with the Protein Preparation Wizard, which adds missing atoms and optimizes the H-bond network by assigning tautomer/ionization states, sampling water orientations, and flipping Asn, Gln, and His residues in the plane of their π-systems. The Gln198 was protonated to Glh198. Finally, restrained energy minimizations were conducted to conclude the system preparation. Ligand 3D structures were sketched manually and transformed into low-energy 3D structures using LigPrep version. Note that the residue numeration in this structure differs from some older versions by two, i.e., Leu253 is equal to Leu255 in some of the previously used docking study structures. Docking calculations to place compounds into the tubulin ligand binding domain were performed with Glide under default parameters; we increased the accuracy of the XP docking mode with an enhanced (2×) sampling option. 

Induced fit docking (IFD) [36] is implemented in the Schrodinger suite and is a protein−ligand docking method that accurately accounts for both ligand and receptor flexibility by iteratively combining rigid receptor docking (Glide) with protein structure prediction (Prime) techniques. While traditional rigid-receptor docking methods are useful when the receptor structure does not change substantially upon ligand binding, success is limited when the protein must be “induced” into the correct binding conformation for a given ligand. Due to the high flexibility of the tubulin binding pocket and expected different binding modes, this method is suitable for this study. The IDF was also performed using the default settings except that we increased the docking accuracy to XP docking mode or enhanced the sampling option. This was done to investigate more precisely other docking poses when using a docking method where the flexibility of specific residues could be considered.

The MD simulations were performed with Desmond v. 7.5 software as it is integrated in Schrodinger suite. The systems were solvated in SPC water molecules with a buffer of 5 Å for the complex and 10 Å for the solvent simulations. The full systems were relaxed and equilibrated using the default Desmond relaxation protocol. This protocol involves energy minimization with restraints on the solute, followed by 12 ps simulations at 10 K using an NVT ensemble, and an NPT ensemble. Subsequently, the restrained system was equilibrated at room temperature using the NPT ensemble. In addition, a 240 ps room-temperature NPT ensemble simulation was performed. Finally, two distinct stability production simulations in the NPT ensemble were carried out, each lasting 500 ns.

The IDF-MD [37] combines several technologies, including pharmacophore analysis, watermaps calculations, docking, rescoring by different scoring functions, molecular dynamics, metadynamics and energy-guided protein structural modeling into a self-consistent and highly computationally efficient approach to predict protein–ligand binding modes within a potentially significantly flexible and dynamic protein binding site such is the case of tubulin. This novel methodology in detailed retrospective and prospective testing succeeded in determining protein–ligand binding modes with a root-mean-square deviation (RMSD) within 2.5 Å in over 90% of cross-docking cases. For comparison, the IFD can identify a pose under 2.5 Å RMSD in the top 2 in only 60% of these cases and in the single top ranked pose in only 50% of cases. The regular docking can achieve only 30–40% success rates in the binding mode prediction. This is especially true in the case of flexible and large binding pockets, thus we consider the use of only simple docking for searching the binding mode of novel compounds in tubulin binding pockets as not practical. In this study, the default protocol of IFD-MD, as implemented in Schrodinger suite 2023-3, was employed. In short, this protocol is separated as follows: at the outset, we employed a ligand pharmacophore analysis, utilizing the template ligand as a reference conformation. This analysis’ purpose is to generate a large and diverse number of possible target ligand binding modes within the ligand binding site of the protein. Further, protein side chains that clash with the pharmacophore-docked ligand positions were refined to produce alternative protein conformations.

The next step involved the utilization of Glide rigid receptor docking to place the ligand within the pre-cataloged alternative protein conformation. Subsequently, energy-guided relaxation of the protein side chains was performed and redocking of the ligand into each of these computationally determined trial protein–ligand binding modes, each with a self-consistently determined docking score and protein structural modeling energy value, calculated using the standard Prime energy model. Next, these trial protein–ligand binding modes were grouped together based on mutual RMSD (root mean square deviation). The top 20 representatives from these clusters, selected for their favorable docking scores and protein structural modeling energy values, were subjected to a comprehensive rescoring process. The detailed rescoring of these 20 trial binding modes began with each binding mode being subjected to 500 ps molecular dynamics simulation and grand canonical water equilibration. Ten separate trial MD runs were generated, and the assessment of each run was based on the evaluation of the last MD frame using the WScore scoring function. Following this, the equilibrated binding modes were progressed into metadynamics simulations, where a gradually increasing force assessed the local stability of the trial binding mode. Finally, the results of the above analysis steps, the best WScore obtained in the 500 ps equilibration runs, and two metadynamics metrics evaluating pose displacement and hydrogen bond persistence, were combined with the Prime energy and Glide docking scores, as well as a number of other empirical terms, to yield a composite scoring function that provided the final ranking of the candidate poses. 

The absolute protein–ligand binding free-energy perturbation (ABFEP) provides a theoretically more rigorous and accurate description of protein–ligand binding thermodynamics [38]. Recently, the ABFEP method has been applied to eight series of closely related compounds that bind to eight different protein receptors. These compound series encompass both neutral and charged ligands, and the results of this benchmarking study have been detailed [38]. The calculated binding free energies have been correlated with experimental results with a weighted average of R^2^ = 0.55 for the entire dataset. An overall RMSE of 1.1 kcal/mol after shifting the zero-point of the simulation data to match the average experimental values has been observed. Based on these results, and also the ABFEP protocols integrated recently in other software [39,40], it can be concluded that this is currently the best method for ranking the compounds and assaying their binding affinity. This conclusion is also supported by our internal tests of many ligand–protein complexes (unpublished data). In the case of inflexible binding sites, we observed a difference in the experimental and ABFEP calculated free energy, which is close to or less than 1 kcal/mol, a value typical for the relative FEP calculations. For more flexible binding sites this difference is typically ~2 kcal/mol. Thus, we decided to assay the ABFEP capability in the case of tubulin and to calculate the free energy of binding for the most promising compound **7c**. 

The ABFEP protocol employed in this study closely resembles the originally proposed double decoupling scheme. The binding of the ligand to the protein receptor is decomposed into a few alchemical steps. First, starting from the physical ligand in water, the van der Waals (vdw) and electrostatic interactions within the ligand and between the ligand and water were slowly removed until the ligand became a dummy. Second, the dummy ligand was connected to the protein’s binding pocket through a set of cross-link restraints, a concept akin to what was initially suggested by Boresch. In the third step, the intraligand and ligand–environment vdw and electrostatic interactions for the restrained ligand were slowly added to the protein binding pocket and the cross-link restraints were relaxed.

In our calculations, we employed the default ABFEP protocol; however, to achieve better sampling we increased the simulation time to 8 ns and added a salt with a concentration of 150 mM. All simulations were performed with Schrödinger Release 2023–3 and OPLS4 force field. Note that this is one of the most advanced force fields currently available and other force fields cannot be used alongside with FEP+ protocol. In each ABFEP simulation, the protein–ligand complex was solvated in an orthorhombic SPC water box. The buffer width was 5 Å. The systems were then relaxed by a series of short molecular dynamic (MD) relaxations, including (1) 100 ps Brownian Dynamics at 10 K with solute heavy atoms restrained (force constant 50 kcal/mol/Å^2^) to relieve minor steric clashes; (2) 12 ps NVT simulation at 10 K with solute heavy atoms restrained; (3) 20 ps μVT simulations where water molecules were sampled via Grand Canonical Monte Carlo (GCMC) at 300 K with solute heavy atoms restrained to equilibrate the water box and solvate the binding pocket; and (4) 20 ps GCMC μVT simulation at 300 K with protein backbone heavy atoms restrained. 

During the GCMC equilibration stages, we set the excess chemical potential of water to −6.137 kcal/mol, and the density of bulk water was maintained at 0.03248 Å–3. After every 50 fs of MD, we attempted GCMC sampling, involving 34,000 trials of water insertion or deletion. (5) Following this relaxation phase, we conducted a 1 ns GCMC μVT simulation at 300 K, during which the heavy atoms of the protein backbone were restrained. After relaxation, a 1 ns GCMC μVT simulation at 300 K was performed with protein backbone heavy atoms restrained. In this simulation, GCMC sampling was attempted every 4.8 ps of MD. 

To identify the optimal set of atoms for the protein–ligand cross-link restraints we conducted an analysis of the interactions between them during the 1 ns MD relaxation. We specifically focused on identifying the atoms involved in the most frequently occurring hydrogen bond and salt-bridge interactions throughout the MD trajectories. These interactions, which were observed in multiple frames of the MD simulations, were documented, and the frequencies of their occurrence were collected.

Following the MD relaxation and the identification of atoms for the cross-link restraints, we selected a representative structure from the MD trajectory as the input for the subsequent FEP simulations. To select this representative structure, we calculated the mean value of each rotatable bond in the ligand based on the samples obtained during the MD trajectory, and the representative structure had the ligand torsions closest to the corresponding mean values. For the complex FEP we employed a total of 68 and 108 λ windows and the charges of ligand atoms were turned off linearly in the first 22 windows. Subsequently, the vdw interactions were turned off, following a nonlinear schedule, across the remaining 46 windows. The three dihedral restraints of the cross-link restraints between the ligand and protein were turned on in a quadratic schedule in the first 34 windows, while the two angle restraints and one distance restraint were turned on in a quadratic schedule in the last 34 windows. To enhance the sampling of ligand conformations and ensure comprehensive coverage, we turned off torsions associated with rotatable bonds (with the exception of those within a ring structure) linearly in all 68 windows.

For each replica, the same equilibration steps as above were used except that all restraints were removed in the last GCMC relaxation stage. Following the equilibration phase, we conducted a production simulation using replica exchange and GCMC water sampling. In this stage, replica exchanges were attempted every 1.2 ps, and the GCMC water sampling protocol remained consistent with the one previously detailed. Each replica was simulated for a duration of 5 ns. The Bennett acceptance ratio method was employed to calculate the free energies between adjacent λ windows, and these values were then summed to determine the overall free energy change for the entire transformation. 

In the solvent FEP calculations, the ligand was extracted from the previously chosen representative structure. It was then solvated within an SPC water box with a 10 Å buffer width. Counterions and additional salt ions at a concentration of 150 mM were introduced into the system. The alchemical ion approach was implemented, particularly for charged ligands, to accurately account for the changes in charge states. A total of 60 λ windows were used for these calculations. The charges of the ligand atoms were gradually turned off in the first 30 windows, employing an empirical nonlinear schedule. Additionally, vdw interactions were selectively turned off in a nonlinear manner across the last 30 windows to account for changes in non-bonded interactions. Same as the complex FEP, the torsions of rotatable bonds except those in a ring were turned off linearly in the entire 60 windows. To prepare each simulation replica for the FEP calculations, a series of equilibration steps were carried out: (1) 100 ps Brownian Dynamics at 10 K with ligand heavy atoms restrained (force constant 50 kcal/mol/Å2); (2) 12 ps NVT simulation at 10 K with ligand heavy atoms restrained; (3) 12 ps NPT simulation at 10 K with ligand heavy atoms restrained; (4) 24 ps NPT simulation at 300 K with ligand heavy atoms restrained; and (5) 240 ps NPT simulation at 300 K. Once these equilibration steps were completed, the production simulations for replica exchange were initiated in the NPT ensemble with solute tempering. Replica exchanges were attempted at intervals of 1.2 ps, and the production runs for each replica lasted 5 ns. Again, the Bennett acceptance ratio method was used to calculate the free energy of the transformation.

In summary, in this work, we used different than previously developed by us protocols [40,41,42,43] including those that combine aMD and FEP+ calculations [40]. Instead, we adopted a combination of IDF-MD and ABFEP approaches to determine the most likely binding mode of the compounds under investigation.

## 4. Conclusions

Building upon the discourse regarding the repurposing of benzimidazole anthelmintics as tubulin inhibitors in cancer therapy, we conducted a screening of a limited library of benzimidazole compounds containing a piperazine moiety at the C-2 position of the benzimidazole core. The screening aimed to assess their combined potential as both anthelmintics and antineoplastics.

The anthelmintic activity study against isolated *T. spiralis* (ML) revealed that the piperazine-benzimidazoles exhibit mild to excellent larvicidal effects in a time and dose-dependent manner. The compound **7c** showed remarkable activity—92.7% efficacy at a concentration of 100 μg/mL after a 48-h incubation period at 37 °C.

The *in vitro* study of the cytotoxicity effect of benzimidazole derivatives against MDA-MB 231 and U87 MG cell lines showed that IC_50_ values of compounds **7b**, **7d**, and **7c** in both tumor cell lines are remarkably similar and lower compare to ABZ as a control. All tested compounds effectively reduced cell migration in U-87 MG and MDA-MB-231 cells, indicating potential for inhibiting tumor cell migration, a critical factor in cancer progression and metastasis. Compound **7c** outperformed others, significantly reducing migration in both cell lines, making it a promising candidate for further research. 

Furthermore, we conducted molecular modeling using a combination of docking, IFD and IFD-MD methods to identify the potential binding mode, and employed the absolute FEP+ (ABFEP) approach to calculate the binding affinity of the most potent compound, **7c**, which exhibits dual anthelmintic and antineoplastic properties, with tubulin. Our findings revealed a unique binding conformation distinct from those previously observed. Notably, the benzimidazole core of **7c** was found to be positioned much more deeply into the β-tubulin binding site, in an unknown binding cavity and make strong interactions with the protein amino acid residues.

## Figures and Tables

**Table 1:** *In vitro* activity of piperazine benzimidazoles against *Trichinella spiralis* muscle larvae ^a^.

Compound	Concentration 50 (100) µg/mL in µM	Efficacy (%) ^b^ After 24 h		Efficacy (%) ^b^ After 48 h	
		50 µg/mL	100 µg/mL	50 µg/mL	100 µg/mL
**6**	0.171(0.342)	13.5	19.5	14.8	33.8
**7a** ^c^	0.131 (0.263)	26.5	56.8	34.7	63.0
**7b** ^c^	0.129 (0.358)	29.6	64.7	33.5	83.0
**7c** ^c^	0.125 (0.249)	35.8	72.0	41.0	92.7
**7d** ^c^	0.110 (0.219)	37.4	75.8	44.0	88.0
**Albendazole**	0.188 (0.377)	10.7	10.9	14.4	15.6
**Ivermectin**	0.057 (0.114)	45.4	49.2	62.0	78.3

^a^ Control, 100 parasites; ^b^ *p* < 0.05; ^c^ Results are published in [30].

**Table 2:** *In vitro* cytotoxicity of the piperazine benzimidazole derivates against U-87 MG and MDA-MB-231 cell lines.

	MDA-MB-231 Cell		U-87 MG Cells	
Compound	IC_50_ (µM)	95% CI (µM)	IC_50_ (µM)	95% CI (µM)
**6**	102.7	(+309.7, −50.23)	104.7	(+1700, −60.69)
**7a**	74.37	(+192.03, −35.18)	54.86	(+121.34, −20.26)
**7b**	39.78	(+18.11, −9.96)	41.42	(+60.08, −15.49)
**7c**	35.84	(+11.11, −7.11)	42.30	(+12.47, −7.62)
**7d**	34.31	(+1.81, −1.71)	38.29	(+4.99, −4.25)
**Albendazole**	83.1	(+103.7, −33.59)	40.59	(+106.31, −19.92)

The 95% confidence interval is reported in brackets as upper (+) and lower (−) limit value of IC_50_.

## Data Availability

Data are available within the article.
